# Natural Killer Cells Promote Early CD8 T Cell Responses against Cytomegalovirus

**DOI:** 10.1371/journal.ppat.0030123

**Published:** 2007-08-24

**Authors:** Scott H Robbins, Gilles Bessou, Amélie Cornillon, Nicolas Zucchini, Brigitte Rupp, Zsolt Ruzsics, Torsten Sacher, Elena Tomasello, Eric Vivier, Ulrich H Koszinowski, Marc Dalod

**Affiliations:** 1 Centre d'Immunologie de Marseille-Luminy, Université de la Méditerranée, Marseille, France; 2 INSERM (U631), Marseille, France; 3 CNRS (UMR6102), Marseille, France; 4 Max von Pettenkofer Institut für Virologie, Ludwig-Maximilians-Universität München, Munich, Germany; 5 Assistance Publique-Hôpitaux de Marseille and Hôpital de la Conception, Marseille, France; University of Wisconsin-Madison, United States of America

## Abstract

Understanding the mechanisms that help promote protective immune responses to pathogens is a major challenge in biomedical research and an important goal for the design of innovative therapeutic or vaccination strategies. While natural killer (NK) cells can directly contribute to the control of viral replication, whether, and how, they may help orchestrate global antiviral defense is largely unknown. To address this question, we took advantage of the well-defined molecular interactions involved in the recognition of mouse cytomegalovirus (MCMV) by NK cells. By using congenic or mutant mice and wild-type versus genetically engineered viruses, we examined the consequences on antiviral CD8 T cell responses of specific defects in the ability of the NK cells to control MCMV. This system allowed us to demonstrate, to our knowledge for the first time, that NK cells accelerate CD8 T cell responses against a viral infection in vivo. Moreover, we identify the underlying mechanism as the ability of NK cells to limit IFN-α/β production to levels not immunosuppressive to the host. This is achieved through the early control of cytomegalovirus, which dramatically reduces the activation of plasmacytoid dendritic cells (pDCs) for cytokine production, preserves the conventional dendritic cell (cDC) compartment, and accelerates antiviral CD8 T cell responses. Conversely, exogenous IFN-α administration in resistant animals ablates cDCs and delays CD8 T cell activation in the face of NK cell control of viral replication. Collectively, our data demonstrate that the ability of NK cells to respond very early to cytomegalovirus infection critically contributes to balance the intensity of other innate immune responses, which dampens early immunopathology and promotes optimal initiation of antiviral CD8 T cell responses. Thus, the extent to which NK cell responses benefit the host goes beyond their direct antiviral effects and extends to the prevention of innate cytokine shock and to the promotion of adaptive immunity.

## Author Summary

To fight viral infections, vertebrates have developed a battery of innate and adaptive immune responses aimed at inhibiting viral replication or at killing infected cells. These responses include the early production of innate antiviral cytokines, especially interferons α and β (IFN-α/β), and the activation of cytotoxic lymphocytes such as the innate natural killer (NK) cells and the adaptive CD8 T cells. While critical for antiviral defense, cytokine or CD8 T cell responses can be detrimental or even fatal to the host when deregulated. Therefore, we need to better understand how the different arms of antiviral immunity are regulated. In particular, NK cells are proposed to play a protective role in a variety of viral infection in humans, but the underlying mechanisms remain poorly understood. Here, in a mouse model of cytomegalovirus infection, we demonstrate that NK cells prevent an excessive production of IFN-α/β and promote more efficient antiviral CD8 T cell responses. We thus show that NK cells can help promote health over disease during viral infections by regulating both innate and adaptive immune responses. It will be important to examine in humans whether NK cells control innate cytokine production to prevent immunopathology and to promote adaptive immunity against herpesviruses, HIV-1, influenza, or SARS.

## Introduction

The development of antiviral immune responses involves the orchestration of a complex network of innate and adaptive immune cells to promote health over disease. Natural killer (NK) cells, plasmacytoid dendritic cells (pDCs), CD11b and CD8α conventional dendritic cells (cDCs), B cells, and CD8 T cells have all been demonstrated to be important for the generation of protective immunity to various viral infections [1–6]. However, how the antiviral defense as a whole is coordinated, and in particular how the functions of different types of immune cells impact the shaping of the global immune response to viruses in vivo, is not thoroughly understood.

The importance of an efficient NK cell response for the promotion of a favorable outcome to viral infections has been demonstrated in both mice and humans [2,7], and the rapid activation of NK cells after infection is a hallmark of their potency as innate immune system effectors [2]. Increasing evidence supports the idea that optimal coordination of immune responses involves an intricate relationship between NK cells and other innate leukocytes. For example, several reports have documented the importance of NK/DC “cross-talk” for the reciprocal activation of these cell types and in the promotion of antitumor immunity as recently reviewed [3,8]. Others have shown the involvement of macrophages as an intermediary in the activation of NK cells via Vα14*i* NK T cells [9]. NK cells have also been shown to have the capacity to interact with neighboring NK cells as well as T cells to stimulate cellular proliferation [10,11].

pDCs were initially identified in humans and mice based on the unique ability of these cells to secrete enormous amounts of IFN-α/β early in response to viral challenge [12]. pDCs respond in this way as a consequence of their ability to recognize molecular signatures of viruses in a manner that is independent of pDC infection [12,13]. Early during the course of murine cytomegalovirus (MCMV) infection, pDCs are the major producers of interferons α and β (IFN-α/β) [13,14], which are critical for host survival [15,16]. IFN-α/β have direct antiviral effects because they can inhibit viral replication in infected cells as well as convey to noninfected cells defense mechanisms that protect them from viral infection. IFN-α/β also mediate a variety of immunoregulatory functions, either activating or inhibitory [17,18]. They contribute to the proliferation of NK cells and induce them to express functional cytotoxic granules, promote cDC phenotypic and functional maturation, and can help to activate antiviral CD8 T cells. In contrast, depending on the context, IFN-α/β can also compromise the immune response of the host by inhibiting DC differentiation [19] or by directly leading to the attrition of CD8 T cells [20,21]. Indeed, different viruses have recently been suggested to induce a profound immunosuppression in the host by inducing overwhelming levels of IFN-α/β [19]. The importance of the host genotypes in the efficiency of IFN-α/β antiviral functions in the context of the infection with specific viruses are well documented [22,23]. In contrast, it is not known whether naturally occurring differences in the interactions between a virus and its hosts exist that may shape IFN-α/β responses by specifically dampening the immunosuppressive functions of these cytokines that are detrimental to the host and beneficial to the pathogen. The identification of such conditions will help to better understand the physiopathology of viral infection and may lead to the development of innovative treatments to fight these infections.

In the early phase of MCMV infection there is a tight temporal relationship between the activation and exertion of effector functions between NK cells and pDCs because the production of IFN-α/β by pDCs promotes NK cell proliferation and cytotoxicity [13,24]. However, the impact of NK cell functions on the pDC response to MCMV remains unexplored. The goal of this study was therefore to evaluate the effect of efficient NK cell responses on pDC IFN-α/β production and on the development of the adaptive immune responses of the host.

C57BL/6 mice are able to mount NK cell responses that can control MCMV replication very efficiently, due to their expression of the Ly49H activating receptor, which endows NK cells with the ability to recognize and kill viral-infected cells expressing the viral ligand m157 (reviewed in [1]). Although the NK cells from BALB/c and other strains of mice lacking the Ly49H gene are strongly activated for the acquisition of the cytotoxic machinery and for the production of IFN-γ early after MCMV infection, they fail to recognize and kill infected cells and can therefore be considered inefficient. Inbred mouse strains that are resistant or sensitive to MCMV infection in an NK cell–dependent manner differ in many other immune parameters, including major histocompatibility (MHC) haplotype as well as DC subset frequency and function [25]. Therefore, rigorous evaluation of the impact of NK cell responses on pDC and adaptive responses requires comparison between mice of the same genetic background differing only in the ability of their NK cells to control the infection. For this, we took advantage of the fact that the Ly49H/m157 activation axis is sufficient for NK cell control of MCMV when introduced into the BALB/c genome [26]. The response of pDCs and CD8 T cells to MCMV were therefore compared between BALB/c mice (Ly49H^−^) and animals on the BALB/c background that are congenic for the C57BL/6 natural killer complex and in particular for Ly49H (C.B6-*Klra8^Cmv1-r^*/UwaJ mice, hereafter named Klra8 [27]). This system thus provides a unique model to study antiviral immune responses in vivo in the context of both efficient (Ly49H^+^) and inefficient (Ly49H^−^) NK cell activation, in the absence of any broad defect in NK cell development or activity that may affect the homeostasis or functions of other immune cells. This system allowed us to demonstrate, to our knowledge for the first time, that NK cells accelerate CD8 T cell responses against a viral infection in vivo. Moreover, we identified the underlying mechanism—the ability of NK cells to limit pDC IFN-α/β production to levels not immunosuppressive to the host—which itself results from the early control of viral replication.

## Results

### Early Control of Cytomegalovirus Replication by NK Cells Is Accompanied by Decreased pDC Cytokine Production

Ly49H triggering by m157 is required for the proliferation and accumulation of NK cells between days 3 and 7 after MCMV infection [28]. Therefore, we first used NK cell expansion as an indicator to show that Klra8 and BALB/c mice generate an NK cell response of different quality to MCMV infection (Figure S1A). We next compared the ability of Klra8 and BALB/c mice to control early viral load. Consistent with reported differences in viral load seen between mice lacking or possessing the Ly49H gene [26,29,30], there was a substantially higher viral burden within the spleens of BALB/c mice when compared to those of congenic Klra8 animals (Figure S1B). In addition, NK cell depletion in Klra8 mice increased viral replication to levels similar to those observed in BALB/c animals (Figure S1C). Thus, these data confirm previous reports demonstrating that Klra8 mice mount a strong NK cell response that is able to efficiently control MCMV replication, in contrast to BALB/c animals.

**Figure 1:**
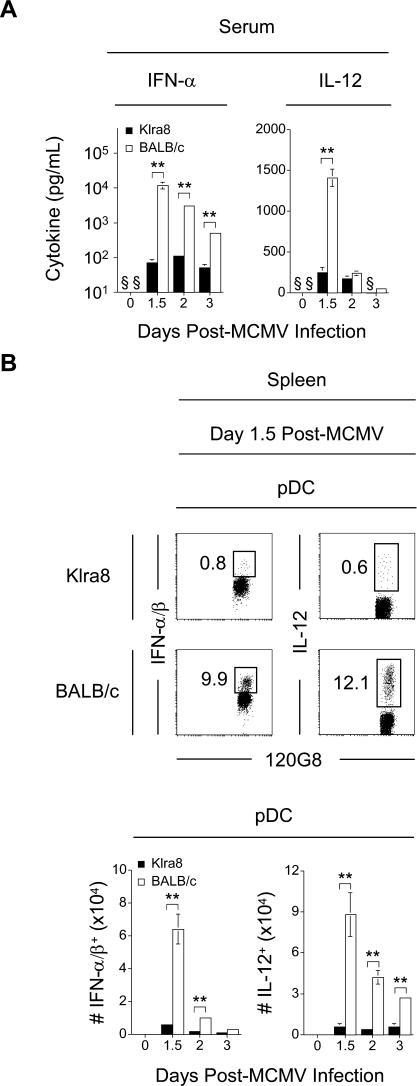
Decreased pDC Cytokine Production in the Presence of Ly49H/m157-Mediated NK Cell Activation (A) Serum from Klra8 and BALB/c mice was collected on days 0, 1.5, 2, and 3 post–MCMV infection. Cytokine levels of IFN-α and IL-12p70 were determined by ELISA. Results are expressed as mean ± SD of three mice per group. (B) Splenic leukocytes were isolated from Klra8 and BALB/c mice and analyzed for the frequency of IFN-α/β^+^ pDCs and IL-12^+^ pDCs directly *ex vivo*. Numbers in dot plots represent the percent of IFN-α/β^+^ and percent of IL-12^+^ cells within the total pDC population. One representative animal from groups of three mice for day 1.5 post–MCMV infection is shown. Graphs represent the total numbers of IFN-α/β^+^ pDCs and IL-12^+^ pDCs present in the spleens of Klra8 and BALB/c mice on days 0, 1.5, 2, and 3 post–MCMV infection. Results are expressed as mean ± SD of three mice per group and in ten thousands of cells. One experiment representative of three for (A, B) is shown. *p ≤ 0.05; **p ≤ 0.01; § = not detected.

To evaluate the impact of efficient antiviral NK cell activity on the induction of IFN-α/β, we first examined the levels of the cytokines present in the serum of Klra8 and BALB/c mice. The serum levels of IFN-α/β were dramatically lower (100-fold) at the peak of the response (day 1.5), and decreased at all other times points examined, in Klra8 mice as compared to BALB/c mice (Figure 1A). The serum levels of other innate cytokines were also significantly lower in Klra8 mice, including IL-12p70 (Figure 1A) and TNF-α (unpublished). Similar results were observed when comparing serum cytokine levels between control-treated and NK cell–depleted Klra8 mice (Figure S2 and unpublished data). Thus, while the systemic production of IFN-α/β and of other innate cytokines in response to MCMV infection is very high in susceptible animals, it is greatly reduced in the presence of an NK cell response that controls viral replication early and efficiently.

In MCMV-infected 129 or C57BL/6 immunocompetent animals, most of the systemic production of IFN-α/β and a significant proportion of that of IL-12p70 comes from pDCs [13,14,31], which are not productively infected [13,32], and does not come from infected cells ([13] and unpublished data). To our knowledge, the contribution of pDCs to IFN-α/β or other innate cytokine production has not been assessed in animals on a BALB/c genetic background, which are the most susceptible to MCMV infection. cDCs infected with MCMV in vitro have been reported to produce high levels of IFN-α/β [33,34], and more generally, any virus-infected cell could theoretically produce these cytokines. Therefore, the increase in the systemic levels of IFN-α/β observed in BALB/c animals could result either from a high activation of pDCs or from a more significant contribution to this function from other cell types or from the high numbers of infected cells in these animals [33,34]. Therefore, we next compared the activation of pDCs for IFN-α/β or IL-12p70 production between BALB/c and Klra8 animals by intracellular staining for these cytokines on *ex vivo* isolated splenocytes (Figure 1B). Less than 1% of the pDCs were found to produce IFN-α/β or IL-12 in Klra8 as compared to 10%–12% in BALB/c mice. Consistent with previous reports on 129 or C57BL/6 animals [14,35], pDCs were the sole producers of IFN-α/β at all the time points examined in BALB/c and Klra8 mice (unpublished data). Both pDCs and cDCs contributed to the production of IL-12 in Klra8 mice. However, the high IL-12p70 serum levels observed in BALB/c mice could solely be attributed to pDCs (Figure 1B and unpublished data). Therefore, a very high activation of pDCs for innate cytokine production was observed in BALB/c mice, which was drastically reduced in congenic animals endowed with efficient antiviral NK cell functions.

To further link the early viral load and the level of IFN-α/β production by pDCs, we performed infections of BALB/c and Klra8 mice with 5-fold serial dilutions of viral inoculums, leading to challenge doses from 10^3^ to 1.25 × 10^5^ pfu/mouse. Systemic IFN-α titers in these animals were measured by ELISA on serum samples, and the numbers of pDCs producing IFN-α/β by flow cytometry (Figure S2). The results clearly showed that the amount of IFN-α/β secreted by pDCs increases dramatically at high viral dose inoculums in Klra8 mice to levels similar to those observed in BALB/c animals. A 5-fold difference in the virus inoculums from 5 × 10^3^ to 2.5 × 10^4^ pfu/mouse is sufficient to switch on IFN-α/β production by pDCs in Klra8 mice from very low levels to nearly maximal levels similar to those observed in BALB/c animals. At low dose viral infection (10^3^ pfu/mouse), BALB/c mice are still activated for nearly maximal pDC cytokine production. Thus, BALB/c and Klra8 animals are confirmed to exhibit dramatically different levels of pDC IFN-α/β production over more than a 10-fold range of viral inoculums. These data also show that the pDCs of Klra8 mice are able to produce high levels of IFN-α/β under conditions of stimulation with high viral inoculums. Moreover, NK cell depletion in Klra8 mice infected with low virus inoculums was also shown to lead to a dramatic increase in pDC IFN-α/β production, almost to the levels observed in BALB/c animals (Figure S2). Altogether, these data thus show that the ability of the host to control MCMV replication early through NK cell responses limits the activation of pDCs and therefore prevents the induction of very high systemic levels of IFN-α/β and IL-12. These data also demonstrate that the pDCs of Klra8 mice are not intrinsically deficient for IFN-α/β production in response to MCMV infection.

### Early Control of MCMV Replication by NK Cells Prevents the Ablation of cDC and Accelerates the Development of Effector Antiviral CD8 T Cell Responses

High systemic levels of IFN-α/β production have been shown to lead to the ablation of cDCs [19] and to the attrition of both antigen-specific and bystander CD8 T cell populations during infection with lymphocytic choriomeningitis virus (LCMV) [20,21]. We therefore hypothesized that the high systemic levels of IFN-α/β induced during MCMV infection in the context of inefficient NK cell activity could lead to the ablation of cDCs and may impinge on the development of antiviral CD8 T cell responses. Indeed, it has been previously reported that MCMV infection leads to a severe ablation of CD8α cDCs in BALB/c mice that is prevented by efficient antiviral NK cell functions in C57BL/6 animals [36]. However, the cause for this ablation of cDCs in BALB/c animals has not been identified, and the effect of this ablation on the development of antiviral CD8 T cells has not been evaluated. Thus, we next compared the numbers of DC subsets and the activation of antiviral CD8 T cells at different time points after infection between Klra8 and BALB/c mice.

**Figure 2:**
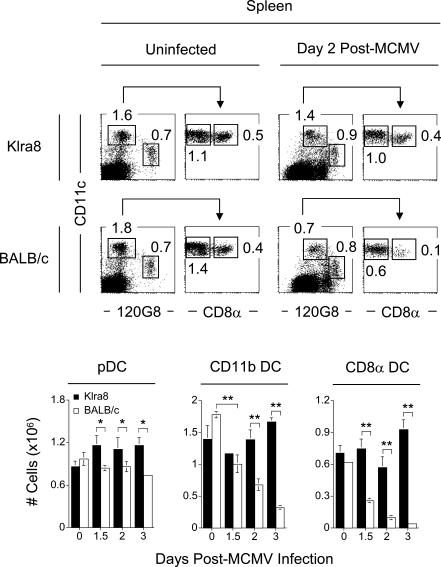
Antiviral NK Cell Responses Prevent Ablation of the cDC during MCMV Infection Splenic leukocytes were isolated from Klra8 and BALB/c mice and analyzed for the frequency of pDCs (120G8^+^ CD11c^int^), CD11b cDCs (120G8^−^ CD11c^hi^ CD8α^−^), and CD8α cDCs (120G8^−^ CD11c^hi^ CD8α^+^) within the DX5^−^ and TCRβ^−^ population. Numbers in dot plots represent percent pDCs, percent CD11b cDCs, and percent CD8α cDCs within the total splenocyte population for one representative animal from groups of three mice for days 0 and 2 post–MCMV infection. Graphs represent the total numbers (in millions) of pDCs, CD11b cDCs, and CD8α cDCs present in the spleens of Klra8 and BALB/c mice on days 0, 1.5, 2, and 3 post–MCMV infection. Results are expressed as mean ± SD of three mice per group. One experiment representative of three is shown. *p ≤ 0.05; **p ≤ 0.01.

We first confirmed that pDC numbers remained at homeostatic levels in BALB/c mice throughout the early phase of MCMV infection, while a severe ablation of cDCs occurred for both the CD11b and CD8α subsets (Figure 2). In contrast, in the presence of an efficient NK cell response, in Klra8 mice, both the cDC and pDC compartments were preserved, consistent with the previous observations reported in C57BL/6 animals [36].

**Figure 3:**
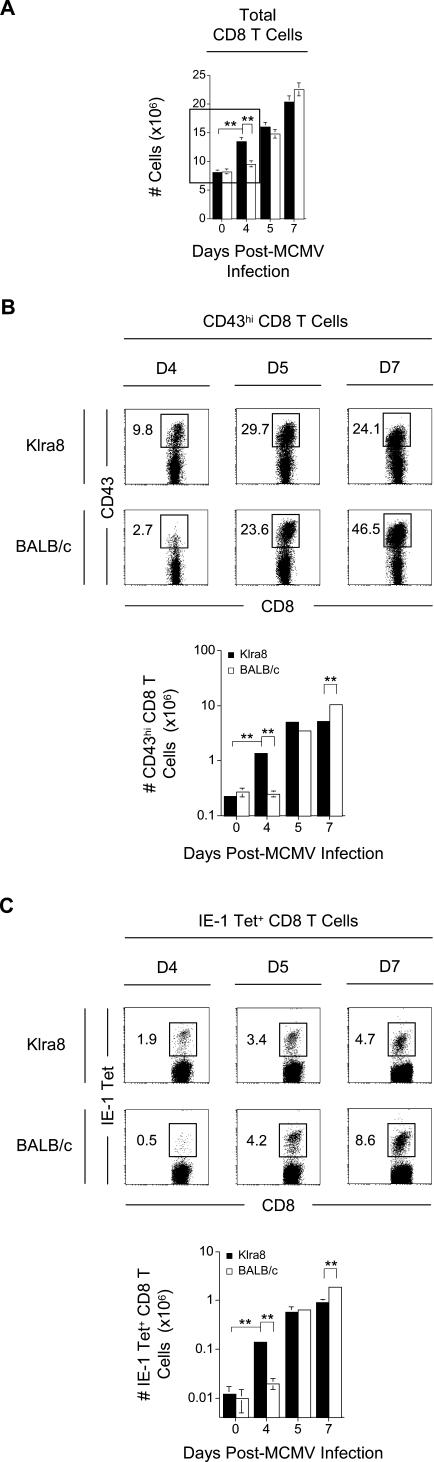
Kinetics of Antigen-Specific CD8 T Cells Expansion in Klra8 and BALB/c Mice in Response to MCMV Infection Splenic leukocytes were isolated from Klra8 and BALB/c mice on days 0, 4, 5, and 7 post–MCMV infection and analyzed for (A) total numbers of CD8 T cells (TCRβ^+^ CD8α^+^), (B) CD43 expression on the total CD8 T cell population, and (C) binding of IE-1-loaded MHC class I tetramers on the total CD8 T cell population. CD43 expression on CD8 T cells and IE-1-loaded MHC class I tetramer binding to CD8 T cells is shown for one representative animal from groups of three mice for days 4, 5, and 7 post–MCMV infection. Numbers in dot plots represent percent CD43^hi^ or percent IE-1 tetramer^+^ CD8 T cells within the total CD8 T cell population. Graphs represent the total numbers (in millions) of CD8 T cells, CD43^hi^ CD8 T cells, or IE-1 tetramer^+^ CD8 T cells. Results are expressed as mean ± SD of three mice per group. One experiment representative of four for (A–C) is shown. **p ≤ 0.01.

We next compared the kinetics of CD8 T cell expansion between Klra8 and BALB/c mice (Figure 3A). We observed a significant increase in total CD8 T cells in the spleen at day 4 post-infection in Klra8 mice but only later in BALB/c animals. We investigated whether this accelerated expansion of CD8 T cells was a result of a faster activation of antiviral effector CD8 T cells. We first analyzed total splenic CD8 T cells for the expression of an O-glycosylated, activation-associated isoform of CD43. The 1B11 monoclonal antibody (mAb) specifically recognizes this isoform of CD43 on CD8 T lymphocytes [37] and identifies the subset of effector cells capable of IFN-γ production and potent cytolytic activity *ex vivo* during the acute phase of a variety of viral infections [38–42]. We observed the appearance of CD43^hi^ effector CD8 T cells starting at day 4 post-infection in Klra8 mice but only later in BALB/c animals (Figure 3B). We then used MHC class I tetramers loaded with a peptide derived from the IE-1 protein of MCMV in order to quantify the subset of antiviral CD8 T cells specific to this epitope (Figure 3C). We observed a faster expansion of the anti-IE-1 CD8 T cells in Klra8 mice, similar to what was observed for total or effector CD8 T lymphocytes. Similar results were obtained when analyzing the CD8 T cell response against another viral peptide, derived from protein m164 (unpublished data). Conversely, and in accordance with what has been previously reported in C57BL/6 mice depleted of NK cells by antibody treatment [43], we observed accumulation of effector CD8 T cells in BALB/c mice at later time points after challenge.

**Figure 4:**
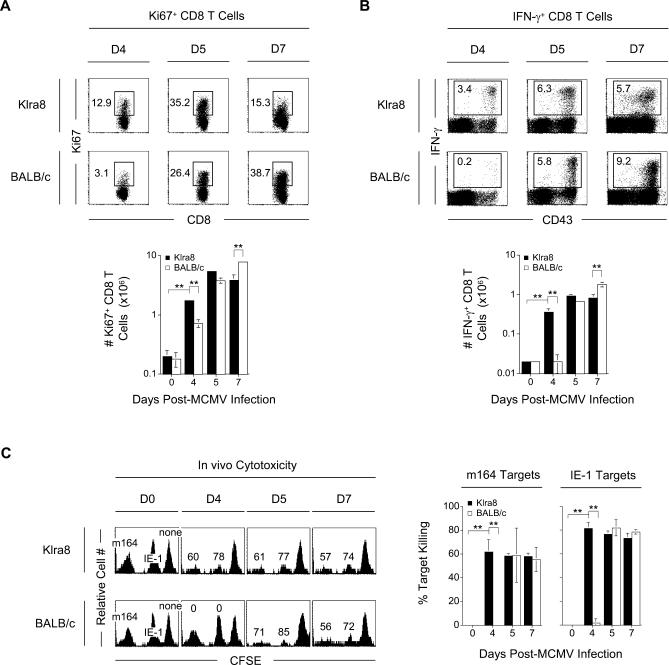
Accelerated Acquisition of CD8 T Cell Effector Functions in Klra8 Mice in Response to MCMV Infection Splenic leukocytes were isolated from Klra8 and BALB/c mice on days 0, 4, 5, and 7 post–MCMV infection. (A) The CD8 T cell population was analyzed directly *ex vivo* for intracellular expression of Ki-67. Intracellular expression of Ki-67 is shown for one representative animal from groups of three mice for days 4, 5, and 7 post–MCMV infection. Numbers in dot plots represent percent Ki-67^+^ CD8 T cells within the total CD8 T cell population. Graphs represent the total numbers (in millions) of Ki-67^+^ CD8 T cells. (B) The CD8 T cell populations were analyzed for their ability to produce IFN-γ after antigen-specific re-stimulation with IE-1 peptide-pulsed P815.B7 cells. Intracellular expression of IFN-γ is shown versus surface CD43 expression for the total CD8 T cell population for one representative animal from groups of three mice. Numbers in dot plots represent percent IFN-γ^+^ CD8 T cells within the total CD8 T cell population. Graphs represent the total numbers (in millions) of IFN-γ^+^ CD8 T cells. (C) The CD8 T cell populations were analyzed for their ability to kill IE-1 and m164 peptide-pulsed target cells in vivo. Target cells were transferred into mice at the time points indicated and the spleens harvested 4 h later. Histograms are gated on PKH26^+^ target cells. Numbers represent the percentage of target cells killed for one representative animal from groups of three mice. Graphs represent the percentage of target cells killed. Results in graphs are expressed as mean ± SD of three mice per group for (A–C). One experiment representative of three for (A, B), and two for (C) are shown. **p ≤0.01.

In order to investigate the effector potential of the MCMV-specific CD8 T cells observed at day 4 post-infection in Klra8 mice, we measured different parameters associated with the protective functions of antiviral CD8 T cells, namely their proliferation, by monitoring the expression of the marker Ki-67 (Figure 4A), their capacity to produce IFN-γ in response to antigen-specific restimulation in vitro using intracellular staining (Figure 4B) and ELISPOT (Figure S3), and their cytotoxic potential by measuring antigen-specific target cell killing in vivo (Figure 4C). For each of these parameters, we observed an accelerated acquisition of effector functions by CD8 T cells from Klra8 mice when compared to BALB/c animals.

**Figure 5:**
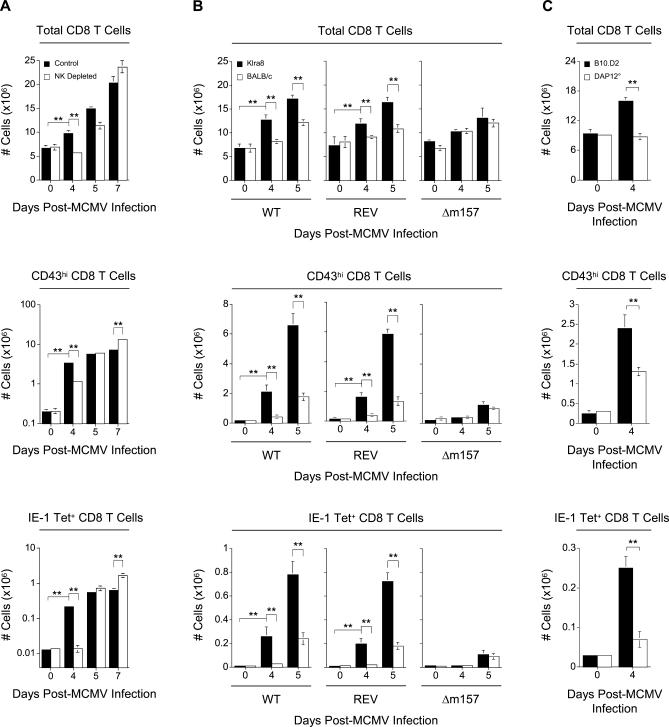
Kinetics of Antigen-Specific CD8 T Cell Expansion in Response to MCMV Infection in Mice Lacking NK Cells or Ly49H-Mediated NK Cell Activation Splenic leukocytes were isolated at the time points indicated from (A) Klra8 mice and Klra8 mice depleted of NK cells infected with MCMV, (B) Klra8 and BALB/c mice infected with in vitro–generated BAC-derived wild-type (WT), revertant (REV), or Δm157 viruses, and (C) B10.D2 and B10.D2-DAP12° mice infected with MCMV. Total CD8 T cell numbers, CD43 expression on CD8 T cells, and IE-1-loaded MHC class I tetramer binding to CD8 T cells were analyzed for (A–C). Graphs represent total numbers (in millions) of cells for each parameter as indicated. Results are expressed as mean ± SD of two (A) or three (B, C) mice per group. One experiment representative of two for (A–C) is shown. **p ≤ 0.01.

As Klra8 and BALB/c mice differ for the whole NK cell locus, which includes genes expressed in several leukocyte subsets, we sought to ensure that the differences observed in the activation kinetics of CD8 T cells between these two mouse strains i) were not due to intrinsic differences in the reactivity of CD8 T cells and ii) were dependent on Ly49H-mediated NK cell activation. This was achieved by showing that no accelerated expansion of CD8 T cells occurs in Klra8 animals i) when the antibacterial responses of Klra8 and BALB/c mice are compared during Listeria monocytogenes infection (Figure S4), or ii) in response to MCMV infection when Klra8 mice are depleted of NK cells (Figure 5A), or when the CD8 T cell responses of Klra8 and BALB/c mice are compared during infection with a Δm157 virus as opposed to infection with wild-type or revertant viruses (Figure 5B). Moreover, the impact of efficient NK cell activity on the development of antiviral CD8 T cell responses was confirmed in mice of another genetic background, as a delay in antiviral CD8 T cell activation was observed in B10.D2 animals deficient for Ly49H-mediated NK cell activation due to the inactivation of the associated adaptor molecule DAP12 (B10.D2.DAP12°), as compared to wild-type controls (Figure 5C). Altogether, these data show that efficient NK cell responses to a viral infection accelerate the development of effector antiviral CD8 T cell responses.

### The Ability of Efficient NK Cell Activity to Optimally Promote Antiviral cDC and CD8 T Cell Responses Can Be Overridden by Type I Interferons

**Figure 6:**
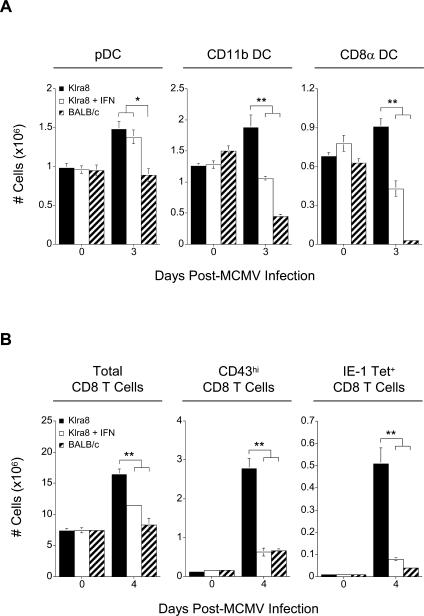
IFN-α-Driven Loss of cDCs and Delay in CD8 T Cell Activation (A) Splenic leukocytes were isolated on days 0 and 3 post–MCMV infection from Klra8, Klra8 treated with IFN-α at 30 and 48 h post-infection, and BALB/c mice. The total numbers (in millions) of pDCs, CD11b cDCs, and CD8α cDCs were determined and are presented as in Figure 2. (B) Splenic leukocytes were isolated on days 0 and 4 post–MCMV infection from Klra8, Klra8 treated with IFN-α at 30 and 48 h post-infection, and BALB/c mice. Total numbers (in millions) of CD8 T cells, CD43^hi^ CD8 T cells, and IE-1 tetramer^+^ CD8 T cells were determined and are presented as in Figure 3. One experiment representative of two for (A, B) is shown. *p ≤ 0.05; **p ≤ 0.01.

To determine whether the dramatic reduction of pDC IFN-α/β production by NK cell functions in Klra8 mice could in part account for the ability of these mice to preserve an intact cDC compartment and to mount early CD8 T cell responses, we examined whether exogenous administration of the cytokines in these animals could directly impact the initiation of cDC and CD8 T cell responses despite efficient NK cell–mediated control of viral load. MCMV-infected Klra8 mice were treated with recombinant mouse IFN-α as described in Materials and Methods, and their DC or CD8 T cell compartments were compared to those of control-treated Klra8 and BALB/c mice (Figure 6A). The treatment of Klra8 mice with IFN-α indeed led to a drastic reduction in both CD11b and CD8α splenic cDCs, but not in pDCs, when compared to their control-treated Klra8 counterparts. Likewise, IFN-α treatment induced a delay in the expansion of total, CD43^hi^, and IE-1 Tet^+^ CD8 T cell numbers (Figure 6B). Of note, the IFN-α treatment administered to the Klra8 mice did not significantly change the level of viral replication in these animals. Very low but detectable levels of infectious viral particles were observed in both control-treated and IFN-α-injected Klra8 mice (1.94±0.35 versus 1.92±0.11 log pfu/spleen), which contrasted sharply with the high viral replication observed in BALB/c animals (5.2±0.04 log pfu/spleen). These results demonstrate that exogenous injection of IFN-α in Klra8 mice is sufficient to decrease the numbers of cDCs and to ablate early antiviral CD8 T cell responses, and, therefore, that excessive levels of IFN-α can have a direct negative impact on antiviral immune cell responses in a manner that is independent of the level of viral replication in the host. The possibility remains that other innate cytokines, such as IL-12 and TNF-α, which are produced at much higher levels in BALB/c as compared to Klra8 mice, may also bear some contribution to this function, in a synergistic or redundant manner with IFN-α/β. In any case, our data strongly suggest that the ability of Klra8 mice to preserve an intact cDC compartment and to mount early CD8 T cell responses is in part due to their ability to control viral replication very early without the need for the host to produce high systemic levels of IFN-α/β. Altogether, our data thus identify how naturally occurring differences in the interactions between a virus and its host can tilt the balance between the various functions of IFN-α/β, and eventually other innate cytokines, towards conditions promoting the induction of early adaptive immunity, rather than the development of a state of transient immunosuppression.

### Early Control of Viral Load by Drug Treatment in BALB/c Mice Recapitulates the Effects of the NK Cell Activity of Klra8 Animals on DC and CD8 T Cell Responses

We next aimed at further understanding the link between NK cell functions, IFN-α/β production, and downstream effects on DCs and CD8 T cells. As BALB/c mice have a much higher viral burden after infection with MCMV, it seemed plausible that the difference in viral load between Klra8 and BALB/c mice could have an impact on the intensity of the DC and CD8 T cell responses. In order to test the impact of the extent of viral replication in vivo on pDC cytokine production and on antiviral CD8 T cell activation, independently of the function of NK cells, we sought a strategy to control viral replication efficiently in BALB/c mice with a tightly controlled timing and magnitude. To achieve this, we utilized an MCMV (DN-SCP-MCMV) that is genetically engineered so that its in vivo replication can be effectively arrested by the administration of doxycycline [44].

**Figure 7:**
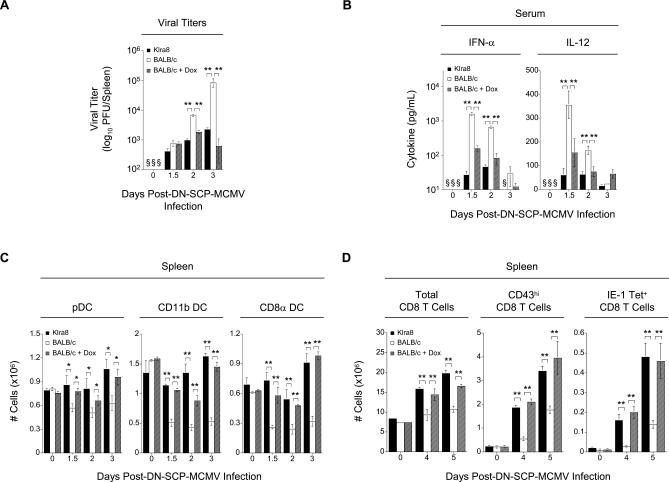
Control of Early Viral Load Results in Decreased pDC Cytokine Production, the Maintenance of cDCs, and the Accelerated Expansion of Antigen-Specific CD8 T Cells (A) Spleens from Klra8, BALB/c, and BALB/c mice treated with doxycycline 20 h post-infection were harvested on days 0, 1.5, 2, and 3 post–DN-SCP-MCMV infection and homogenized. Viral titers were determined and are presented as in Figure S1B. (B) Serum from Klra8, BALB/c, and BALB/c mice treated with doxycycline 20 h post-infection was collected on days 0, 1.5, 2, and 3 post–DN-SCP-MCMV infection. Cytokine levels of IFN-α and IL-12p70 were determined and are presented as in Figure 1. (C) Splenic leukocytes were isolated from on days 0, 1.5, 2, and 3 post–DN-SCP-MCMV infection from Klra8, BALB/c, and BALB/c mice treated with doxycycline 20 h post-infection. The total numbers (in millions) of pDCs, CD11b cDCs, and CD8α cDCs were determined and are presented as in Figure 2. (D) Splenic leukocytes were isolated on days 0, 4, and 5 post–DN-SCP-MCMV infection from Klra8, BALB/c, and BALB/c mice treated with doxycycline 20 h post-infection. Total numbers (in millions) of CD8 T cells, CD43^hi^ CD8 T cells, and IE-1 tetramer^+^ CD8 T cells were determined and are presented as in Figure 3. One experiment representative of two for (A–C), and four for (D) are shown. *p ≤ 0.05; **p ≤ 0.01; § = not detected.

Using this system, we were able to design a doxycycline treatment protocol which, in BALB/c mice, closely mimics the extent and the kinetics of efficient NK cell–mediated control of viral replication observed in Klra8. Indeed, under the experimental conditions selected, the viral burdens in Klra8 mice and in DN-SCP-MCMV-infected, doxycycline-treated BALB/c mice were comparable at the different time points examined, and much lower than in untreated DN-SCP-MCMV-infected BALB/c animals (Figure 7A). The reduction in viral burden in doxycycline-treated DN-SCP-MCMV-infected BALB/c animals led to a significant, strong decrease in the serum levels of the pDC-derived cytokines IFN-α and IL-12p70 (Figure 7B) and allowed the maintenance of cDCs (Figure 7C). Moreover, we observed an accelerated activation of antiviral CD8 T cells in doxycycline-treated, as compared to untreated, DN-SCP-MCMV-infected BALB/c mice. The magnitude and kinetics of expansion of antiviral CD8 T cells in doxycycline-treated DN-SCP-MCMV-infected BALB/c mice was comparable to that which is observed in the presence of the efficient antiviral NK cell response of Klra8 mice (Figure 7D). The treatment of DN-SCP-MCMV-infected Klra8 mice with doxycycline had no effect on any of the parameters tested (unpublished data). Thus, our data indicate that, in Klra8 mice, the ability of NK cells to dampen innate cytokine production by pDCs and thus to promote optimal conditions for the initiation of antiviral cDC and CD8 T cell responses result from their exquisite capacity to control viral replication early and efficiently after infection.

## Discussion

The results from this study demonstrate that efficient NK cell responses promote the accelerated generation of effector antiviral CD8 T cells during infection in vivo, in part by preventing the generation of very high, immunosuppressive levels of antiviral cytokines. Thus, our study demonstrates that NK cells can serve not only as effector lymphocytes but also as a regulator of immune system function for defenses against viral infections. Therefore, our data bring important and original advances to the understanding of the contributions NK cells make to immunity against infectious disease, by demonstrating that i) they control the functions of another critical player in the innate antiviral responses, the pDC, and modulate the cytokine milieu induced early after challenge, and ii) they accelerate the generation of effector antiviral CD8 T cells. Other mechanisms through which NK cell activities can modulate immune responses to pathogens have been summarized in several recent reviews [8,45,46] and include in vivo i) the regulation of the homeostasis and of the maturation of DCs and ii) the prevention of a detrimental persistence of the activation of the CD8 T cells at later time points during the immune response. Very recently, it was also shown that perforin-mediated NK cell killing down-modulates the activation of macrophages and prevents the development of a hemophagocytic lymphohistiocytosis-like syndrome during MCMV infection [47].

Our results demonstrate that NK cells can dramatically decrease the intensity and duration of pDC activation by controlling viral burden, which prevents the production of very high systemic levels of IFN-α/β, and eventually other innate cytokines, that can have detrimental effects for the host. We show that this mechanism also protects against the MCMV-mediated loss of splenic cDCs [36]. It has been reported that that both measles virus and LCMV can exploit the host's IFN-α/β response to inhibit cDC development and drive cDC loss in vivo [19]. Our results suggest that MCMV also can induce high production of IFN-α/β to promote its own survival by ablating cDCs and delaying the activation of antiviral effector CD8 T cells. In light of this observation, it is interesting to note that MCMV has developed strategies to actively counteract the antiviral responses to IFN-α/β or IFN-γ within infected cells [48], as opposed to the mechanisms employed by negative-strand RNA viruses, which act to shut down the production of these cytokines by infected cells [49] or pDCs [50]. Indeed, even though complete deficiency in IFN-α/β responses is associated with a dramatic increase in the susceptibility of mice to MCMV infection [16], it clearly appears that the benefit of high level IFN-α/β production for the host is less than that brought by an efficient NK cell response (since Klra8 mice show viral titers that are 1,000-fold lower than those seen in BALB/c mice, even though Klra8 mice produce 100-fold less IFN-α/β). Thus, it is tempting to speculate that the efficient NK cell activity driven by the Ly49H activating receptor and its ability to dampen pDC IFN-α/β production and to promote adaptive immunity is a direct host countermeasure to the subversion of the IFN-α/β response by MCMV. Altogether, our results suggest that the NK cell response governs the balance between the positive and negative effects of IFN-α/β, and eventually other innate cytokines, for the optimal orchestration of the immune response to MCMV.

Our data indicate that efficient NK cell activity contributes to the adaptive arm of the immune response to MCMV by promoting the accelerated expansion of antigen-specific CD8 T cells. Like the contribution NK cells make to the maintenance of the cDC compartment, our data support a role for NK cell control of pDC IFN-α/β production for the promotion of antigen-specific CD8 T cell expansion. IFN-α/β can have both positive and negative effects on CD8 T cell responses [20,21,51–53]. For example, the optimal expansion of CD8 T cells in response to LCMV infection has been demonstrated to be dependent on the ability of the CD8 T cell compartment to receive IFN-α/β-mediated signals [53]. However, within the same viral system, IFN-α/β also drives the early attrition of both antigen-specific and bystander CD8 T cell populations [20,21]. Here, we show that the delay in the expansion of antiviral effector CD8 T cells in the absence of an efficient NK cell response to MCMV occurs within the context of excessive production of IFN-α/β by pDCs. Moreover, we demonstrate that exogenous administration of IFN-α can override the ability of efficient NK cell activity to promote the accelerated expansion of functional antiviral CD8 T cells. The IFN-α-mediated effects on CD8 T cell expansion could be direct or a downstream consequence of the loss of cDCs. However, our results suggest a direct activity of IFN-α/β on CD8 T cells, as the administration of IFN-α leads to the complete disappearance of early antiviral CD8 T cells but only to an incomplete decrease in cDC numbers.

Although not the focus of this study, our data also show that delayed CD8 T cell responses reach higher levels and are sustained for longer periods of time in the absence of efficient antiviral NK cell activity, which is consistent with previous observations [43]. This extended activation of the T cell compartment is required for MCMV control under these conditions and likely results from the poor ability of the innate immune system to control the virus early. One could hypothesize that this could also lead to chronic inflammation and long-term detrimental effects for the host, including the increased susceptibility of BALB/c mice to MCMV-induced T cell–dependent autoimmune diseases such as myocarditis [54]. Thus, efficient NK cell responses could be beneficial to the host not only by early direct antiviral effects but also by reducing the degree of antiviral T cell activation required for later control of viral replication and therefore the indirect costs this may bear for health.

To our knowledge, this study is the first to demonstrate how naturally occurring, genetically determined differences in the interactions between a virus and its host can tilt the balance between the various functions of IFN-α/β towards conditions promoting the induction of early adaptive immunity rather than towards the development of a state of transient immunosuppression. Our data also confirm that NK cell antiviral functions prevent the generation of a chronic state of CD8 T cell activation later during the infection that may otherwise lead to immunopathology. A key question for future studies will be to determine how general this function of NK cells is for antiviral defense. Such mechanisms may also be in place during HIV infection where genetic [55] or functional [56–58] evidences suggest a role in the variation of NK cell functions for resistance or susceptibility to the development of AIDS, where detrimental effects of excessive levels of IFN-α/β [59] or of pro-inflammatory cytokines [60,61] have been recently suggested, and where the initiation of antiviral CD8 T cell responses appears delayed [62–64]. This could also be the case for infections with highly pathogenic strains of influenza, as excessive production of pro-inflammatory cytokines have been shown to occur and proposed to play a crucial role in immunopathology [65,66], while NK cells can confer resistance through recognition of infected cells by the NKp46 receptor [67]. Very recently, researchers have demonstrated that human NK cells are able to recognize DCs infected with the influenza or Ebola viruses [68,69], while a correlation between unregulated IFN-α/β responses and a malfunction of the switch from innate to adaptive immunity has been reported during fatal SARS [70], which further highlight the potential relevance of our observations to a variety of viral infections. Thus, it will be important to determine the impact of NK cell function on the modulation of innate and adaptive immunity and on the development of immunopathology in other models of viral infection. Moreover, our results imply that in individuals susceptible to such viral infections, pharmacological control of viral replication very early should benefit them not only by directly limiting viral cytopathogenicity, but also by establishing conditions better suited to the development of balanced, protective immunity, since this is the case in mice susceptible to MCMV infection when viral replication is controlled by drug treatment.

A role for NK cells in the generation of antitumor CD8 T cell responses has been linked to their ability to increase inflammation via secreting IFN-γ and promoting IL-12 production by DCs [71,72]. Collectively, these studies and ours reveal a role for NK cells as mediators of an “innate cytokine balance” for the optimal generation of the immune response, whereby NK cells are required to produce cytokines to increase inflammation when it is intrinsically low (during tumor development) and to prevent excessive production of innate cytokines when it can be intrinsically high (in the context of pathogenic encounters). Of note is the apparent requirement, in both instances, for NK cells to engage in cognate receptor–mediated interactions to naturally develop immunoregulatory functions. For example, during the response to MCMV, the systemic activation of NK cells by IL-12 to produce IFN-γ in a Ly49H^−^ environment is not sufficient to promote their control of viral replication and their associated immunoregulatory functions. These cognate interactions could also enable NK cells to act as direct regulators of antiviral immunity, as already illustrated for the generation of antitumor CD8 T cell responses. During the response to MCMV, Ly49H expression by NK cells dramatically increases the efficiency of recognition and killing of infected cells. Ly49H may also enable NK cells to deliver IFN-γ in a proper place and time to help the priming of antiviral CD8 T cells in infected mice through a tripartite interaction with MCMV-infected m157-expressing DCs. The importance of these cognate interactions for the promotion of efficient NK cell functions may differ depending on the tissues and on the target cell types, as exemplified by the heightened requirement for Ly49H-dependent mechanisms for MCMV control in the lungs [73].

In conclusion, we demonstrate that efficient NK cell activity contributes to the optimal orchestration of innate and adaptive immunity during the course of a viral infection in vivo. The implications of these findings extend to the design of therapeutic strategies to fight human disease in two ways, as they emphasize i) that the benefits of innate cytokine production for immune cell recruitment and activation can be outweighed by detrimental effects that can directly reduce the capacity of the host to fight infection, as well as ii) the need to better understand and take into account the complex interactions of relatively rare cell types that occur in a physiological context for the purpose of exploiting the human immune system to promote health over disease.

## Materials and Methods

### Mice.

BALB/c mice (Charles River Laboratories, http://www.criver.com/) were purchased for use in these studies. C.B6-*Klra8^Cmv1-r^*/UwaJ, referred to as Klra8, (Jackson Laboratory, http://www.jax.org/), B10.D2, and B10.D2-DAP12-deficient (DAP12°) animals were bred in pathogen-free breeding facilities at the Centre d'Immunologie de Marseille-Luminy (Marseille, France). Experiments were conducted in accordance with institutional guidelines for animal care and use. Protocols have been approved by the French Provence ethical committee (number 04/2005) and the US Office of Laboratory Animal Welfare (assurance A5665–01).

### In vivo treatment protocols.

Stocks of wild-type Smith Strain MCMV salivary gland extracts [14] and bacterial artificial chromosome (BAC)-derived wild-type, DN-SCP-MCMV [44], Δm157-MCMV, and m157-revertant were prepared as previously described [73]. Infections were initiated on day 0 with the i.p. delivery of 5 × 10^3^ PFU (for in vivo–derived virus) or 2 × 10^5^ PFU (for in vitro–derived virus). These modest doses were chosen because they do not induce lymphopenia in infected animals who harbor leukocyte numbers equal to or greater than those of uninfected controls (not shown). In addition, these doses are likely to be closer to the physiologic doses reached upon natural exposure to the virus through contact with infected animals. For the in vivo arrest of DN-SCP-MCMV replication, 200 μg of doxycycline was delivered by i.p. injection 20 h post-infection followed immediately by the addition of 2 mg/ml doxycycline plus 5% sucrose in the drinking water. 50,000 units of recombinant mouse IFN-α (HyCult Biotech, http://www.hbt.nl/) was administered by i.p. injection at 30 and 48 h post–MCMV infection. This dose was titrated in ELISA and shown to correspond to 25 ng of the ELISA IFN-α standard. Thus, since the cytokine titers measured by ELISA in the serum of BALB/c mice at 36 h post-infection range between 5 and 10 ng/ml, and the volume of the lymph and blood of an adult mouse can roughly be estimated around 5 ml, the dose of rmIFN-α injected in Klra8 mice should be similar to the physiologic levels of the cytokine naturally induced in infected BALB/c mice. NK cells were depleted by delivery of 100 μg of purified anti-NK1.1 mAb (PK136) on days −1 pre–MCMV infection, and days 1 and 3 post–MCMV infection. Control mice were treated with 100 μg of mouse IgG (Jackson ImmunoResearch Laboratories, http://www.jacksonimmuno.com/). Uninfected mice were depleted of NK cells on the schedule of day 4 infected mice. To assess NK cell depletion, a combination of anti-DX5 and anti-TCRβ staining was used in order to avoid the risk of underevaluating the numbers of remaining NK cells, as may have occurred due to a potential problem of epitope masking if the antibody used for immunophenotyping had been the same as the one used for depletion. NK cell depletions were greater than 98% (Figure S5).

### Isolation of lymphocytes.

For the analysis of CD8 T cell populations, spleens were minced, passed through nylon mesh and washed in PBS, 5 mM EDTA, and 3% FCS (PBS/EDTA/FCS). For the analysis of DC populations, spleens were digested by collagenase (liberase CI; Boehringer Mannheim, http://www.roche.com/) and teased apart by repeating pipeting in PBS/EDTA/FCS. In both protocols, erythrocytes were osmotically lysed by ammonium chloride treatment. Thereafter, cell suspensions were kept in PBS/EDTA/FCS unless specified otherwise. Total live splenocytes were counted by trypan blue exclusion using a hemocytometer. Total numbers of specific leukocyte subsets were calculated for each individual mouse as (percent of these cells in the live gate of total splenocytes) × (total live splenocyte numbers).

### CD8 T cell stimulation for IFN-γ production.

For intracellular IFN-γ detection, splenic lymphocytes were isolated and then incubated with IE-1 (^168^YPHFMPTNL^176^) or m164 peptide (^257^AGPPRYSRI^265^) (10^−7^ M) pulsed P815.B7 cells at a 10:1 ratio for 6 h with brefeldin A (Sigma-Aldrich, http://www.sigmaaldrich.com/) added for the last 3 h of culture. Cells were then harvested and analyzed for CD8α, CD43, and intracellular IFN-γ protein by three-color staining followed by flow cytometry.

### In vivo cytotoxicity assay.

Antigen-specific CD8 T cell–mediated cytotoxicity was assayed as described in [74]. Briefly, splenocytes from naive mice were costained with PKH26 (Sigma-Aldrich) and either 1 μM, 100 nM, or 1 nM CFSE (Molecular Probes, http://probes.invitrogen.com/). Labeled cells were then pulsed with the indicated peptides, mixed in equal ratios, then transferred i.v. (5 × 10^6^ total cells) into the indicated groups of mice. Lymphocytes were isolated from the spleens of recipient mice 4 h post-transfer and analyzed for PKH26 (all transferred cells) and level of CFSE expression (unpulsed or peptide-pulsed cells). Percent killing within the PKH26^+^ gate was calculated by: 100 − ([(% peptide-pulsed in infected/% unpulsed in infected)/(% peptide-pulsed in uninfected/% unpulsed in uninfected)] × 100).

### Quantification of viral titers and serum cytokine levels.

Spleens were homogenized [14], and viral titers were determined by plaque assay using mouse embryonic fibroblasts with centrifugal enhancement or NIH-3T3 cells [73]. Serum was collected at the indicated time points and cytokine levels were determined by ELISA for IFN-α (PBL Biomedical Laboratories, http://www.interferonsource.com/) and IL-12p70 (R&D Systems, http://www.rndsystems.com/) per the manufacturer's instructions.

### Abs and reagents.

DX5-PE, CD43-PE (clone 1B11), CD11c-PE (clone HL3), CD8α-PerCp (clone 53–6.7), TCR-β-allophycocyanin (clone H57–597), IFN-γ-allophycocyanin (clone XMG1.2), IL-12-allophycocyanin (clone C15.6), and streptavidin-PE were purchased from BD Pharmingen (http://www.bdbiosciences.com/). CD8α-FITC (clone CT-CD8α) was purchased from Caltag (http://www.caltag.com/). Ki-67-FITC (clone MM1) was purchased from Novocastra (http://www.vision-bio.com/). Purified rat anti-mouse IFN-α (clone F18 and RMMA-1) and anti-IFN-β (clone RMMB-1) were purchased from TEBU-Bio (http://www.tebu-bio.com/). 120G8 mAb was provided by Schering-Plough (http://www.schering-plough.com/) and conjugated to Alexa Fluor-488 using a kit from Molecular Probes. Isotype controls for each mAb were purchased from the appropriate manufacturer. The following tetramers conjugated to PE were obtained through the NIH Tetramer Facility (Atlanta, Georgia, United States): H-2L(d)/IE-1(^168^YPHFMPTNL^176^) and H-2D(d)/m164(^257^AGPPRYSRI^265^). The above-mentioned reagents were used for FACS analysis in this study.

### Flow cytometric analysis.

Cells were first incubated with 2.4G2 mAb for 20 min. Cells were then stained with mAbs specific for cell surface markers or isotype controls for 30 min at 4 °C. Cells were then washed and fixed in 2% paraformaldehyde in PBS. Intracellular staining for Ki-67, IFN-γ, and IL-12 was performed using the Cytofix/Cytoperm kit (BD Pharmingen). Intracellular staining for IFN-α/β was performed as previously described [35]. Depending on the experiments, 2.5 × 10^5^ to 2 × 10^7^ events were collected on a FACSCalibur. The data were acquired and analyzed using CellQuest software (BD Biosciences). Isotype controls were used to set gates for the presented FACS analyses.

### Statistical analyses.

Statistical analyses were performed in Microsoft Excel 5.0 (Microsoft Corporation, http://www.microsoft.com/) using Student's two-tailed *t* tests. Mean ± standard deviation (SD) was calculated for each graph. If visually absent, error bars are too small to be depicted based on the scale of the *y*-axis.

## Supporting Information

Figure S1 Klra8 Mice but Not BALB/c Animals Show a Dramatic Expansion of NK Cells during MCMV Infection and Control Early Viral Replication Efficiently through NK Cell Activity (437 KB TIF)

Figure S2 Impact of High Dose and Low Dose MCMV Infections, and of NK Cell Depletion, on Systemic IFN-α/β Production in Klra8 Mice (864 KB TIF)

Figure S3 Accelerated Acquisition of CD8 T Cell Effector Functions in Klra8 Mice in Response to MCMV Infection (502 KB TIF)

Figure S4 Kinetics of Antigen-Specific CD8 T Cell Expansion in Klra8 and BALB/c Mice in Response to L. monocytogenes Infection (385 KB TIF)

Figure S5 Assessment of the Efficiency of NK Cell Depletion (223 KB TIF)

## Abbreviations

BAC: bacterial artificial chromosome
cDC: conventional dendritic cell
DC: dendritic cell
Klra8: C.B6-*Klra8^Cmv1-r^*/UwaJ
mAb: monoclonal antibody
LCMV: lymphocytic choriomeningitis virus
MCMV: murine cytomegalovirus
NK: natural killer
pDC: plasmacytoid dendritic cell
